# COVID-19 and Networks

**DOI:** 10.1007/s00354-021-00134-2

**Published:** 2021-09-10

**Authors:** Tsuyoshi Murata

**Affiliations:** Department of Computer Science, School of Computing, Tokyo Institute of Technology, W8-59 2-12-1 Ookayama, Meguro, Tokyo 152-8552 Japan

**Keywords:** Network science, Epidemics, Influence maximization, Temporal networks

## Abstract

Ongoing COVID-19 pandemic poses many challenges to the research of artificial intelligence. Epidemics are important in network science for modeling disease spread over networks of contacts between individuals. To prevent disease spread, it is desirable to introduce prioritized isolation of the individuals contacting many and unspecified others, or connecting different groups. Finding such influential individuals in social networks, and simulating the speed and extent of the disease spread are what we need for combating COVID-19. This article focuses on the following topics, and discusses some of the traditional and emerging research attempts: (1) topics related to epidemics in network science, such as epidemic modeling, influence maximization and temporal networks, (2) recent research of network science for COVID-19 and (3) datasets and resources for COVID-19 research.

## Introduction

The global outbreak of the new coronavirus infection (COVID-19), which began in early 2020, brought a profound impact on the research community. Especially in urban areas, it has been impossible to gather at universities and research facilities, which are the hubs of research.

To overcome this difficulty, research related to COVID-19 is being conducted in various fields. The number of publications on COVID-19 in the world has been growing exponentially, which is unique compared to the increase in the number of publications in past infectious disease cases such as SARS in 2002.

In response to the global crisis brought about by COVID-19, an unprecedented level of research activity is being undertaken. In addition to medicine and pharmacology, artificial intelligence can also be expected to contribute a lot to COVID-19.

The process of human contact can be modeled as a network (graph), and by focusing on its structure, we can elucidate and control the phenomena in infectious diseases. In this paper, we will mainly discuss topics such as infection models, influence maximization problem, and temporal networks from the perspective of network science, and then introduce some examples of network science research on COVID-19, machine learning workshops, and data and resources for COVID-19 research.

## Infection Models in Network Science

### Infection Models

Networks and the epidemiology of directly transmitted infectious diseases are fundamentally linked. Early epidemiological models were based on population wide random-mixing, but in practice each individual has a finite set of contacts to whom they can pass infection. Therefore, knowledge of the structure of the network is necessary to compute the epidemic dynamics [1]. Empirical social contact patterns are essential to understand the spread of infectious diseases. There are large-scale population surveys of contacts of real societies [2], which will be the basis of the epidemiological models. The spread of COVID-19 in Hong Kong is investigated based on contact tracing data to characterize clusters and superspreading [3]. As is often pointed out, the structure of human interaction networks is known to be scale-free, and the structure can be used for predicting the spread of the virus and the strategies for mitigation [4]. A recent paper [5] indicates that the observed linear growth of the number of COVID-19 patients cannot be explained by the existing epidemic models, and propose contact networks with a critical number of social contacts.

In a network structure in which people (vertices) are connected by contact and communication (edges), consider a model in which each vertex is in one of the following states

- S (Susceptible)
- I (Infected)
- R (Recovered or Removed)

Depending on what state each vertex takes and how it transitions, there are variations of models such as SI model, SIR model, and SIRS model. The SI model is based on $S \rightarrow I$ transition, the SIR model is based on $S \rightarrow I \rightarrow R$ transition, and the SIRS model is based on $S \rightarrow I \rightarrow R \rightarrow S$ transition.

In the SIR model, for example, let us suppose the proportions of the number of people in each of the *S*, *I*, and *R* states are *s*, *x*, and *r*, respectively. Then they can be expressed by the following differential equations

1 $$\begin{aligned} \frac{\mathrm{{d}}s}{\mathrm{{d}}t}= & {} -\beta s x \end{aligned}$$

2 $$\begin{aligned} \frac{\mathrm{{d}}x}{\mathrm{{d}}t}= & {} \beta s x - \gamma x \end{aligned}$$

3 $$\begin{aligned} \frac{\mathrm{{d}}r}{\mathrm{{d}}t}= & {} \gamma x \end{aligned}$$

4 $$\begin{aligned} s + x + r= & {} 1, \end{aligned}$$

where $\beta$ and $\gamma$ are the parameters that represent the ease of state transition. The former is the degree to which the infected (*I*) infects the surrounding uninfected (*S*), and the latter is the degree to which the infected (*I*) becomes the recovered (*R*). By solving this, we get the following equations

5 $$\begin{aligned} s= & {} s_{0}e^{\frac{-\beta r}{\gamma }} \end{aligned}$$

6 $$\begin{aligned} r= & {} 1 - s_{0}e^{\frac{-\beta r}{\gamma }}, \end{aligned}$$

where $s_{0}$ is the value of *s* in the initial state. In the early stage of infection, $s_{0} \approx 1$ since the number of infected people is negligible. Then we get the following equation

7 $$\begin{aligned} r = 1 - e^{\frac{-\beta r}{\gamma }}. \end{aligned}$$

If we set $c=\beta /\gamma$, we get the following equation for *r*

8 $$\begin{aligned} r = 1 - e^{- c r} . \end{aligned}$$

It is obvious that there is a solution $r=0$ in Eq. (8), but in addition to that, if $c>1$, *r* also has a positive solution, while if $c \le 1$, it does not have a positive solution. In general, it is difficult to find the value of this positive solution analytically, so it is solved numerically. Since $c = \beta /\gamma$, where $\beta$ represents the ease of state transition of $S \rightarrow I$ and $\gamma$ represents the ease of state transition of $I\rightarrow R$, we can conclude that $S \rightarrow I$ is more likely to occur than $I\rightarrow R$ when $c = \beta /\gamma > 1$. As time goes by, the number of vertices in state *I* increases and it becomes an epidemic. When $c \le 1$, there is no epidemic. The Basic Reproduction Number ($R_{0}$) is an epidemiological indicator of how many people an infected person can spread the disease to. In this SIR model, $R_{0} = \beta /\gamma$. In other words, an epidemic occurs when one infected person spreads the disease to one or more other people.

When simulating the change in the number of infected people over time based on such an infection model, the necessary parameters are as follows. The change in the number of infected people over time is highly dependent on these parameters.

- $\beta$: the ease of state transition of $S \rightarrow I$
- $\gamma$: the ease of state transition of $I \rightarrow R$
- $x_{0}$: ratio of infected people in the initial state
- network: the structure of human contacts

The value of $\beta$ is determined by the nature of the virus of the infectious disease, the availability of vaccines, and the way people behave. The value of $\gamma$ is determined by the nature of the virus of the infectious disease, the medical system, and the availability of therapeutic drugs. The value of $x_{0}$ is unknown in many cases (if it were clearly known, it would likely be possible to restrain the infected person at that point). The network structure of contacts among people is determined by the way people behave.

**Fig. 1:**
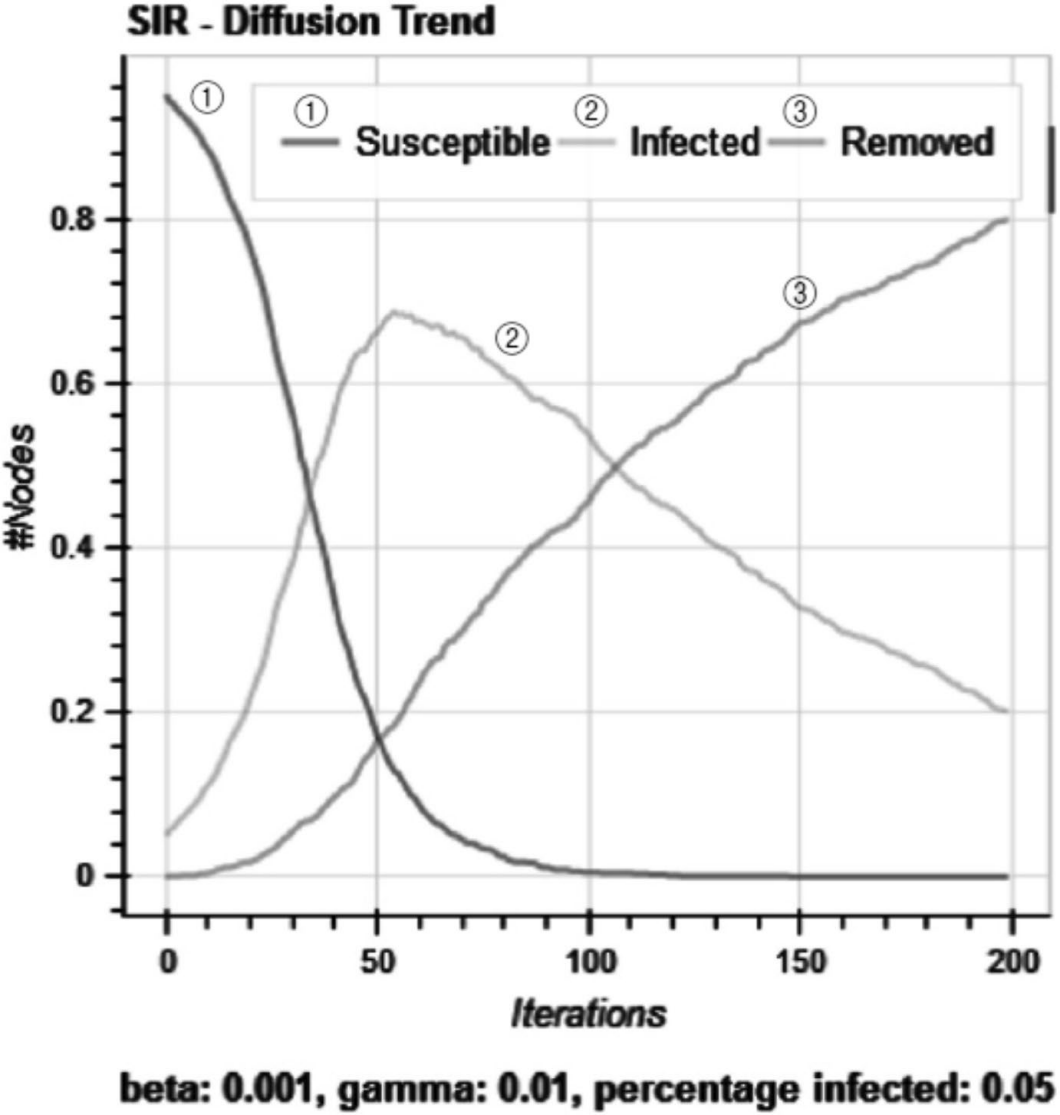
An example of NDlib simulation result of SIR model

As an example, a NDLib (described below) simulation result of the SIR model for a random network with $\beta = 0.001$, $\gamma = 0.01$, and $x_{0} = 0.05$ is shown in Fig. 1. As shown in the figure, the number of infected (*I*) increases and then decreases in this case. Depending on the parameters and network structure, the simulation results can vary greatly.

In the above discussion, the ease of state transition of $S \rightarrow I$ is expressed only in terms of the parameter $\beta$, but more elaborated information diffusion models are as follows [6].

- IC (Information Cascade)
- LT (Linear Threshold)

In the former case, each infected person selects one of the neighboring uninfected people around him and tries to infect the neighbor only once, but whether he gets infected or not depends on the parameters of the edge connecting the two. In the latter case, each vertex has a threshold for the ease of infection, and if the number of infected people around a person exceeds the threshold, then the person becomes infected.

By increasing the number of states and parameters in the model, it may be possible to model more complex temporal changes, but on the other hand, the estimation of parameter values corresponding to actual infections also becomes more complex.

Tools for simulating changes in the number of infected people over time include the Python-based NDLib (Network Diffusion Library) [7] which was developed as a result of the EU’s Horizon 2020 project, and the Scratch Epidemic Simulator from MIT Media Lab [8].

### Influence Maximization Problem

The influence maximization problem is as follows: “Given a network, an information diffusion model, and a constant *k*, find a set of vertices of size *k* such that the disease or information spreads the most from the *k* vertices”. Although it is the exact opposite of the vaccination problem [9], which is to find the vertex set to be quarantined to prevent the spread of infection, it is thought to provide guidelines for preventing the spread of infection, and many studies have been conducted.

There are various definitions of network centrality, such as degree centrality, eigenvector centrality, Katz centrality, PageRank, betweenness centrality, and so on. Centrality has been the subject of much research in network science [10]. Determining the effective centrality for influence maximization problem is non-trivial. Furthermore, even if individual vertices are diffusing to many of their surroundings, as a set they may not be diffusing to many other vertices because their neighbors may overlap with each other.

The influence maximization problem has been proven to be NP-hard [11] and it is difficult to compute an exact solution in large networks. Therefore, Monte Carlo simulations and approximate solutions using heuristics have been studied [12–16].

### Temporal Networks

In most of the previous discussions, it was assumed that the network structure does not change over time. However, in real social networks, contacts and communications are rarely constant, and the network structure usually changes over time. It is not always easy to extend the features of static networks to temporal networks. For example, if Mr. A and Mr. B met yesterday, and Mr. B and Mr. C meet today, the disease could be transmitted from Mr. A to Mr. C, but not the other way around. Temporal networks have a directionality that static networks do not have.

In network science, there has been a great deal of research on representations, models, and dynamics of temporal networks [17–21]. As a method to generate a static network that is equivalent to a temporal network in terms of disease transmission, there is a study that showed that an exponential-threshold network with weights that decay exponentially with time and edges between vertices whose weight sum exceeds a threshold is superior to a network created by a simple sum of contacts between vertices [22]. As a result of simulating which factors of the structure in a temporal network are important, it was found that the beginning and end of contact between vertices and the total number of contacts are important in considering the scale of infection in disease transmission, while the timing of contact between the beginning and end and the distribution of the intervals are less important [23]. In the sentinel surveillance problem of finding vertices that should be observed at fixed points in a network to detect disease outbreaks quickly and accurately, Holme ranked vertices in 38 dynamic and static networks for three evaluation scales (time until disease is detected or disappears, time until disease is detected, and frequency of disease detection). When ranking the vertices in temporal and static networks, it was reported that the rankings differed greatly depending on the evaluation scale, and that (unlike other problems) the results were similar in static and temporal networks [24]. To stop the spread of infectious diseases, it is desirable to focus on isolating people who come into contact with a large number of people or who connect different groups. Many definitions of centrality exist for static networks, and research is being done to extend them to temporal networks, or to give completely new definitions. There have also been studies on extending the influence maximization problem described in the previous section to temporal networks [25, 26], and on community detection in temporal networks [27, 28].

One of the main features of networks is that they can be visualized to give a bird’s eye view of the whole picture, but visualizing temporal networks is more challenging than visualizing static networks. For example, there is a convenient method of dividing the time into hourly segments, grouping the contacts within that hour into a single static network, and then representing the collection as a multilayer network [29], but many issues remain, such as how to determine the granularity of the time.

## Various Approaches for Combating COVID-19

In this section, we will introduce some recent work on network science for COVID-19, related machine learning workshop, as well as data and resources for such research.

### COVID-19 Research Related to Network Science

Ending the global pandemic of COVID-19 will require the implementation of multiple large-scale countermeasures, including social distance, testing, and contact tracing. The most commonly used transmission models are commonly used infection models include the SIR model described above and the SEIR (Susceptible–Exposed–Infected–Recovered) model, which adds exposure (E) to the SIR model. Recently, Giordano et al. [30] have proposed a new model, SIDARTHE, to predict the process of an epidemic. This model considers eight states of infection: susceptible (S), infected (I), diagnosed (D), ailing (A), recognized (R), threatened (T), healed (H), and extinct (E). A unique feature of this model is that it distinguishes the status of infected persons according to whether they have been diagnosed or not and according to the severity of their symptoms. The authors of the paper argue that the distinction between diagnosed and undiagnosed infected persons is important because diagnosed infected persons are usually isolated and are less likely to spread the infection. The authors compare the simulation results with actual data on the COVID-19 epidemic in Italy to model scenarios of countermeasures that can be implemented. In doing so, the basic reproduction number ($R_{0}$) is changed according to the implementation of measures such as lockdown and social distance. The paper concludes by arguing that social distance is necessary and effective to end the ongoing COVID-19 pandemic, and that extensive testing and contact tracing is necessary to safely exit the lockdown. However, they state that these conclusions are based on several assumptions, and that the limitation of this type of modeling study is that the real-life infection situation is highly dependent on how and when countermeasures are implemented.

Manchein et al. [31] analyze the cumulative increase in the number of COVID-19 cases in countries in Asia, Europe, North America, and South America through March 27, 2020. In infection models such as SIR, the number of infected people increases theoretically exponentially ($y = k^{x}$, where *k* is a constant) depending on the value of the basic reproduction number ($R_{0}$). However, this study claims that (1) power-law growth in the number of infected people ($y = x^{k}$) was observed in all of the nine countries (Brazil, China, France, Germany, Italy, Japan, South Korea, Spain, and the United States) on four continents that were analyzed, (2) by introducing Distance Correlation (DC) in comparing data from two countries, the country-specific power-law curves are statistically highly correlated, suggesting the global universality of these curves, and (3) it is argued that soft quarantine strategies are inefficient in flattening the growth curve of the number of infected people. In this paper, the SEIR model is used as the infection model, and measures are proposed to enable the government to reach a flattening of the power-law curve. In addition to social distance, which is well known to be relevant, the authors suggest that measures to identify and quarantine a large number of infected individuals on a daily basis will help flatten the power-law curve. They argue that the high correlation between the power-law curves of different countries strongly suggests that measures for the containment of COVID-19 can be applied to many countries.

Chinazzi et al. [32] used a global infection transfer model called GLEAM to predict the impact of travel restrictions on the rapid spread of COVID-19 in mainland China, both within China and internationally. The SLIR (Susceptible–Latent–Infected–Recovered) model is used as the infection model. Travel was banned in Wuhan on January 23, 2020, suggesting that there were already many infected travelers in most Chinese cities at that time. This travel ban in Wuhan only delayed the overall epidemic in China by 3–5 days, but had a noticeable effect on the international epidemic, with international movement of infected people decreasing by almost 80% until mid-February, according to the paper. The international movement of infected people also differed greatly before and after the travel ban in Wuhan. Before the ban, the countries with the highest number of infected people moving from Wuhan were Thailand, Japan, Taiwan, South Korea, and Malaysia, but after the ban, the countries with the highest number of infected people moving from Wuhan (mainly from Shanghai and other cities in China) were Japan, Thailand, South Korea, Taiwan, and the United States, in that order. The paper also simulates the spread of the disease by varying the rate of movement of people within China and the rate of travel restrictions between China and other countries. According to the results, simply restricting travel to and from China by 90% is not sufficient to stop the spread of the disease, and must be combined with a reduction in the mobility rate within China of at least 50%. However, these results also depend on parameters such as the basic reproduction number, and the values are determined based on reactivity analysis and background knowledge of other infectious diseases such as SARS.

Forster et al. [33] analyzed the phylogenetic network of 160 complete human COVID-19 genomes published in the GISAID database [34] and found three central variants distinguished by amino acid changes, one of which is an outgroup of bat coronaviruses. They argue that such a phylogenetic network can help track down the as-yet-unknown source of COVID-19 infection, and thereby be used in quarantine to prevent the recurrence of a global epidemic.

Human behavior is an important factor for explaining and controlling disease spread. Block et al. evaluate the effectiveness of three social distancing strategies designed to keep the curve of the number of patients flat. These are: limiting interaction to a few repeated contacts akin to forming social bubbles; seeking similarity across contacts; and strengthening communities via triadic strategies [35]. Meidan et al. propose “alternating quarantine” for sustainable epidemic mitigation [36]: at every instance, half of the population remains under lockdown while the other half continues to be active—maintaining a routine of weekly succession between activity and quarantine. This strategy provides a dramatic reduction in transmission, comparable to that achieved by a population-wide lockdown. The impact of social distancing has been inconsistent, with some regions rapidly nearing disease elimination and others seeking delayed peaks or nearly flat epidemic curves. Nande et al. show based on simulations that long delays between the adoption of social distancing and observed declines in cases, hospitalizations, and deaths occur in many cases [37]. The authors claim that the strength of within-household transmission is a critical determinant of success. Kissler et al. estimate the future of COVID-19 transmission in May 2020 based on seasonality, immunity and cross-immunity for other coronaviruses using time-series data from the United States [38]. According to this research, the key metric for the success of social distancing is whether critical care capacities are exceeded. To avoid this, prolonged or intermittent social distancing may be necessary into 2022.

Northeastern University in the U.S. has the following website, which includes the above mentioned [32] as well as other reports and news about infection modeling and control.

- COVID-19 Research [39]

Other publishers such as Physical Review Journals and AIP Publishing have also published a collection of COVID-19 related papers.

- Coronavirus (COVID-19) Collection (Physical Review Journals) [40]
- Coronavirus (AIP Publishing) [41]

### Workshop

For machine learning approaches, Graph Representation Learning and Beyond (GRL+) workshop [42] was held in July 2020 collocated at ICML 2020 conference. On the website of this workshop, William L. Hamilton of McGill University presented a slide titled “Graph Methods for COVID-19 Response” on how graph representation learning can be used to fight against COVID-19. According to this slide, he discusses biomedical treatment data, epidemiological network data, and supply chain networks as target COVID-19 data, all of which represent heterogeneous relationships. As the applications of using these data, the following five applications are listed.

- Computational drug design
- Computational treatment design
- Epidemiological forecasting
- Demand forecasting and supply chain optimization
- Outbreak tracking and tracing

Computational drug design is the task of finding new molecular structures that work against viruses. Its sub-problems are (1) molecular representation and property prediction and (2) molecular generation and search. For the former, the author describes the use of Graph Neural Networks, but also mentions that it is still an open challenge. For the latter, the author describes two approaches: (1) latent space optimization and (2) search and reinforcement learning.

Computational treatment design is the task of predicting whether a drug that already exists will be effective when repurposed against COVID-19. There are two approaches: a structure-based approach that uses known information about viral protein structures, and a network-based approach that uses knowledge of the biological interactions between drugs, diseases, and proteins. Since the former is similar to the previous computational drug design, preliminary studies on the latter have been presented mainly. In those studies, heuristics and biological domain knowledge are used, and guidelines for drug repurposing using Graph Neural Networks are introduced.

Since the remaining tasks (epidemiological forecasting, demand forecasting and supply chain optimization, and outbreak tracking and tracing) all involve (1) heterogeneous relational data, (2) temporal information and changes, and (3) vertex-level forecasting, spatio-temporal Graph Neural Networks are claimed to be useful. As for epidemiological forecasting and outbreak tracking and tracing, however, the author stated that there are concerns about ethics, privacy, and fairness, and that they should be discussed with the ethics committee of the university first.

On the workshop site, in addition to links to various datasets related to COVID-19, an MIT site called AI Cures is referenced [43]. The site claims to be from a group of machine learning and life science researchers who are collaborating to develop machine learning methods to find promising antiviral molecules for COVID-19 and other pathogens.

### Data and Resources

The AIHub also has a COVID-19 resource page for AI researchers called AIHub [44]. This site contains information about COVID-19, including online meetings held mainly around April 2020, a call for researchers to analyze the infection status, COVID-19 related datasets, research funding information, computational resources, and challenges.

### Free Access to Patented Technologies

The Open COVID Coalition, an international group of scientists and lawyers, has announced the Open COVID Pledge, a call for organizations around the world to make their patents and copyrights freely available to fight COVID-19 [45]. Under the Open COVID Pledge, the patents and copyrights will be made available free of charge to those who wish to use the intellectual property to end the pandemic or minimize the impact of the disease. In response to this call, major companies such as Facebook, Amazon, IBM, Microsoft, and Uber have agreed to freely use hundreds of thousands of patents held by these companies for COVID-19 related development for a limited period of time [46]. major U.S. universities such as Harvard, MIT, and Stanford are also seeking to open their intellectual property to the public free of charge in other ways. This is a desirable move, at least in the short term.

Among Japanese universities, Tokyo Institute of Technology has launched “Social Rebooting Technology Initiative” [47] to plan and implement social contributions and support for the promotion of related research in light of the recent social situation triggered by the spread of COVID-19. With the aim of contributing to society quickly, mainly through existing research, and to play a role in guiding social cooperation toward more serious research and development efforts, Tokyo Institute of Technology is offering 131 patents owned by the university free of charge for a certain period of time.

## Concluding Remarks

We are now being hit with the n-th wave of case numbers in the COVID-19 tsunami and the first wave of books about it [48, 49]. As the COVID-19 infection situation and responses to it are constantly changing, there is a possibility that this paper may not cover everything that needs to be covered or may miss the mark by the time it is published.

Investigations into the spread of the Severe Acute Respiratory Syndrome (SARS) epidemic of 2002–2003 have revealed the existence of a super spreader, a patient who became the source of infection that spread to many people [50]. The scale of infection and the number of people moving internationally are very different between SARS and COVID-19. It remains to be seen what lessons can be learned from COVID-19 for the future, and how network science can contribute to these lessons.
